# Early Debridement, antibiotics and implant retention (DAIR) in patients with suspected acute infection after hip or knee arthroplasty - safe, effective and without negative functional impact

**DOI:** 10.7150/jbji.39168

**Published:** 2019-12-10

**Authors:** Luís Henrique Barros, Tiago Amorim Barbosa, João Esteves, Miguel Abreu, Daniel Soares, Ricardo Sousa

**Affiliations:** 1 Department of Orthopaedics, Centro Hospitalar Universitário do Porto, Porto, Portugal.; 2 Department of Infectious Diseases, Centro Hospitalar do Porto, Porto, Portugal.; 3 Grupo TrofaSaude - Hospital em Alfena, Valongo, Portugal.; 4 GRIP (Porto Bone and Joint Infection Unit), Centro Hospitalar Universitário do Porto, Portugal.

**Keywords:** acute prosthetic join infection, antibiotics, debridement, irrigation, retention, functional outcome

## Abstract

**Introduction:** Debridement, antibiotics and implant retention (DAIR) is known to be effective in treating acute periprosthetic joint infection (PJI). However, deciding to perform additional surgery in the early postoperative period may be challenging as there is the concern of adding morbidity and clinical presentation is often subtle. We mean to assess the impact of early DAIR on final functional outcome.

**Methods:** A case-control comparison was performed between patients that underwent DAIR for suspected PJI between 2010-2016 and controls randomly selected (1:2 ratio) from a list of primary joint replacements. Patients were matched for anatomic site, age, gender, American Society of Anesthesiologists (ASA) classification, body mass index and follow-up time. The outcome of surgical treatment and complications were assessed and Hip disability and Osteoarthritis Outcome Score (HOOS) or Knee injury and Osteoarthritis Outcome Score (KOOS) were performed.

**Results:** Thirty-eight cases were included at a mean follow-up of 42 months. Infection was not confirmed in one patient. There was one infection related-death and three other cases of treatment failure that required a two-stage revision. Overall success rate was 89.2%. There were no significant patient reported differences regarding final functional outcome between both groups: pain 91±6 vs. 87±13; other symptoms 90±8 vs. 90±9; activities of day living 86±8 vs. 85±14; sport 63±13 vs. 57±16; quality of life 78±17 vs. 76±16.

**Discussion:** These findings support that DAIR for suspected acute PJI is safe, effective and causes no impact on final functional results. Thus, a low threshold for assuming infection and subsequent DAIR may safely be adopted in the early postoperative period.

## Introduction

Prosthetic joint infection (PJI) is one of the most dramatic complications after a total joint replacement with potential devastating long-term physical and psychological consequences, leading to significant impairment in the quality of life. In adition, it is associated with increased costs and burden to the healthcare system 1.

Debridement, antibiotics and irrigation with implant retention (DAIR) is an appealing treatment alternative especially for acute PJI where it seems to offer the best results 2. However, diagnosing early infection can be challenging and deciding to perform additional surgeries in the early postoperative is not always easy as the risk/benefit ratio of further surgery is not always clear. If clinical presentation is clear (e.g. fever, purulent drainage, etc.) no one will doubt to go ahead with DAIR but often clinical presentation of early PJI is much more subtle and the fear of adding morbidity may delay or even preclude the decision.

Persistent wound drainage is a known risk factor for infection 3, 4 but what exactly constitutes abnormal wound drainage is a matter of debate and recommendations on the correct way to manage it vary considerably. To further intricate decision-making, surgeons often face the inherently complex and also controversial issue of differentiating superficial infection requiring superficial wound washout or perhaps antibiotic therapy only from deep surgical site infection requiring a formal DAIR procedure 5.

The goal of this study is to evaluate an institutional policy of “aggressively” pursuing DAIR in the early postoperative period with respect to its accuracy in identifying deep surgical site infection, its effectiveness and safety profile and most importantly to evaluate its impact on final functional outcomes.

## Methods

### Patient selection

This is a retrospective case-control study at a single institution. We included all patients that underwent DAIR with modular parts exchange for suspected acute postoperative infection of total hip or knee arthroplasty between 2010-2016. Our current institutional recommendation is to perform a formal DAIR in the early postoperative period if: 1) persistent wound drainage and C-reactive protein (CRP) rising trend after the initial 72 hours; 2) persistent wound drainage by day 10 regardless of CRP measurements; 3) strong clinical suspicion such as significant wound healing disturbance (i.e. “superficial” wound infection, “superficial” skin necrosis or wound dehiscence) is present at any time in the early weeks postoperatively regardless of CRP measurements.

Controls were randomly selected (1:2 ratio) from a list of primary arthroplasty patients in the same time period matching for anatomic site, age, gender, American Society of Anesthesiologists (ASA) classification, Body Mass Index (BMI) and follow-up time.

### Surgical Treatment Protocol

All patients with suspected acute PJI underwent a formal DAIR with systematic and thorough debridement with mobile parts exchange. The first step is to reopen the wound and clean any subcutaneous fluids and clots. If the deep fascia is found to be closed, then superficial debridement is performed, collecting separate tissue samples for microbiological study. When this layer is macroscopically clean, synovial fluid is collected with a needle, in order to obtain a sample of uncontaminated synovial fluid, and then the deep fascia is open with a new set of instruments. After arthrotomy, the mobile components are removed in order to increase access and allow better cleansing of all prosthetic interfaces. A thorough and meticulous debridement of all devitalized tissues and extensive synovectomy are performed. At this stage, a minimum of five tissue samples are systematically obtained and sent to microbiological study, preferably choosing macroscopically purulent tissues and/or tissues in intimate contact with the prosthesis. After thorough excision of suspicious tissues and removal of all the suture material, copious irrigation is performed. Three liters of chlorhexidine gluconate scrub are used initially, followed by another three liters of saline solution. After this step, the wound is temporarily involved in sterile compress dressing and the surgical team change gloves and protective clothing and a new set of sterile surgical tools are presented. An additional 1 L of saline irrigation is performed prior to the replacement of new mobile components to replace those that have been removed. In all cases a drain is used. The wound is closed in layers as tightly as possible using a technique similar to primary surgery (absorbable suture in the deep fascia and staples in the skin). No local antibiotics were used.

### Medical Treatment Protocol

When facing a persistent wound drainage or unfavorable wound healing, we do not initiate antibiotic therapy. Serial CRP measurements are performed daily and nonoperative measures such as modification of venous thromboembolism prophylaxis, nutritional supplementation, dressing modification and restriction of range of motion are taken.

As described earlier, if CRP shows a rising trend after the initial 72 hours, there is persistent wound drainage after day 10 or a strong clinical suspicion arises a decision to go ahead with surgery is taken.

After surgery and microbiology samples are gathered, broad spectrum intravenous antibiotics are initiated (i.e. vancomycin and piperacillin/tazobactam in accordance with our local microbial flora antibiotic susceptibility patterns).

As soon as definitive microbiology results are available, antibiotic therapy is deescalated according to isolated pathogens(s) and antibiotic susceptibility patterns. Every case was considered on an individual basis and in a multidisciplinary consultation with an infectious diseases specialist. Switch from intravenous to oral regimens is not dependent on any previously established time, but a combination of good clinical response with and the possibility of antibiotic(s) with good oral bioavailability. Antibiotic therapy was usually extended for at least three months. Whenever possible an “anti-biofilm” antibiotic combination, including rifampicin for staphylococci and ciprofloxacin for Gram-negative infections, was used.

### PJI definition, Follow-up and Outcome

Given the paucity of evidence regarding acute PJI definition we chose to define PJI exclusively based on the positivity of at least one deep (subfascial) sample collected intra-operatively either synovial fluid or periprosthetic tissue.

After hospital discharge, all patients were routinely followed up in clinic at about three and six weeks, three, six and twelve months and then annually.

A 2-year minimum follow-up after antibiotic therapy discontinuation was considered, as most failures occur during this time period 6, 7. Infection was deemed to be cured when the wound healed uneventfully with no signs or symptoms of infection, the joint was pain free and serum inflammatory parameters gradually decreased and were persistently low despite antibiotic discontinuation. Diagnoses of a chronic infection or the need for subsequent surgery were considered treatment failures. Medical-surgical complications and re-operation rates were recorded.

Functional outcome was assessed using joint specific patient-reported outcome measures: Hip disability and Osteoarthritis Outcome Score (HOOS) or Knee injury and Osteoarthritis Outcome Score (KOOS). HOOS and KOOS scores are a patient-reported outcome measurement instrument, developed to assess the patient's opinion about their hip and knee and associated problems, respectively. The HOOS is composed of 40 questions and looks at five subscales: Pain (10 items), Symptoms (5 items), Activity of Daily Living (17 items), Sport and Recreation Function (4 items) and Hip-Related Quality of Life (4 items). The KOOS evaluates both short-term and long-term consequences of knee injury. It holds 42 items in five separately scored subscales: Pain, Symptoms, Function in Daily Living, Function in Sport and Recreation (Sport/Rec), and Knee-related Quality of Life (QOL). In both cases, a total score is calculated by using a simple formula to produce a score that ranges from 0-100, with zero representing extreme hip or knee problems and 100 representing no problems.

### Statistical Analysis

All the statistical analysis was performed by SPSS 25.0 software. Univariate analysis was conducted to assess differences of patients-related data and functional scores between cases and controls. Independent samples t-tests for continuous data and Chi-square tests for dichotomous data were performed. P values <0.05 were considered as statistically significant.

## Results

A total of 2503 primary hip or knee arthroplasties were performed during this period. Thirty-nine patients with suspected acute postoperative total hip or knee arthroplasty infection were submitted to DAIR: 4 patients with isolated persistent drainage by day-10, 24 patients with CRP rising trend after initial 72 hours and simultaneously persistent wound drainage or wound healing disturbance and in 11 patients we found strong clinical suspicion as superficial wound infection or wound dehiscence. The primary arthroplasties and DAIR procedures were performed by six surgeons and the DAIR was not always performed by the same initial operating surgeon. Mean time from primary joint replacement to DAIR was 22.6 (6-30) days and mean follow-up time was 42.1 (24-66) months.

A mean of two superficial and two deep tissue samples as well as one synovial fluid sample were collected. Infection was confirmed postoperatively, with most patients showing multiple positive samples and only one case with a single deep positive sample in final microbiology results. Only one patient had no positive cultures and, in this case, antibiotic therapy was interrupted as soon as definitive microbiology results were available.

After surgery, broad spectrum intravenous antibiotics were initiated and then deescalated as soon as definitive microbiology results were available. A mean total antibiotic therapy time of 3.2 (3-4.1) months was found.

DAIR successfully eradicated infection in 34 out of 38 patients (89.5%). No adverse events due to the antibiotics were found. There was one infection-related death occurring as the consequence of a multi-organ failure in the early postoperative period, 3 weeks after a THA, despite surgical treatment and broad-spectrum antibiotics. This was an 83 years-old patient with major comorbidities (i.e. ASA 3, diabetes mellitus and Child B liver failure) in which an enterococcus faecium was isolated. Three other cases progressed to chronic infection requiring subsequent two-stage revision. In these cases, mean time from arthroplasty to DAIR was 24 (23-28) days and mean time for failure was nine weeks. One methicillin-resistant Staphylococcus aureus, one methicillin-sensitive Staphylococcus aureus, and one Staphylococcus epidermidis were isolated in each patient. The same antibiotic protocol was applied in these patients including rifampicin. No other major medical-surgical complications related to the procedure were noted.

Of those who achieved infection eradication, 26 were available for functional evaluation (12 hips and 14 knee patients). Reasons for exclusion of the functional outcome analysis were: two unrelated complications that preclude functional evaluation (one periprosthetic fracture and one contralateral amputation], three patient deaths from unrelated causes (all of them without evidence of infection relapse after at least two years follow-up) and three patients were lost to the follow-up.

Fifty-two matched uneventful joint replacement patients were also assessed. Control group and patients with acute PJI did not differ with respect to joint, age, gender, ASA classification, BMI and mean follow-up time (Table 2).

Patient self-reported functional outcomes after a 2-year minimum follow-up, showed no significant differences between cases and controls in any category including pain and other symptoms, as quality of life (see Table 3). When evaluating the functional outcome by clinical presentation (persistent drainage *vs.* more obvious clinical findings) and by microorganism type (Staphylococcus aureus or even MRSA *vs.* other pathogens) no differences were found.

## Discussion

Debridement, antibiotics and irrigation with implant retention (DAIR) is commonly viewed as a first-line treatment alternative for acute postoperative PJI^2^. Nevertheless, the decision to perform it in the early postoperative period is not always easy. Often, diagnosis of acute periprosthetic infection can be extremely difficult because clinical manifestations are not always obvious. Although acute PJI cases are sometimes obvious presenting fever and purulent discharge, they can also have subtle changes such as small wound dehiscence or prolonged wound discharge 8. Whether these minor changes are cause or consequence of infection is debatable but they are well established risk factors 3, 4.

The optimal management of such unclear cases is also a matter of debate. A recent International Consensus Meeting 9 suggested that surgical intervention should be considered after five to seven days of persistent wound discharge despite conservative measures. It was also suggested that the surgeon should rely on the status of the fascia to decide whether to simply perform a superficial irrigation and debridement and wound closure (if the fascia is judged to be intact) or proceed to modular components exchange if the fascia is not intact 9.

In our department we shave been systematically using a uniform formal DAIR approach to address all cases of suspected acute infection as previously described in the methods. Although it may be controversial, we do not believe in fascia as an effective barrier to the progression of bacteria in such an early postoperative period. A classic paper by Berbari et al. 10 has shown that treatment for a (supposedly) superficial infection is an enormous risk factor for ultimately developing a PJI with an odds ratio of 35.9 (95% confidence interval 8.3 to 154.6).

Using deep fluid and tissues microbiology results to define infection we found that almost all patients (38/39) were culture positive in deep samples. Although it was not a goal of this paper, we have frequently come across cases in which synovial fluid presented seemingly innocent features including low leukocyte counts despite being culture-positive, thus advising caution in interpreting aspiration to rule out deep infection and choose superficial debridement over a formal DAIR as it has been recently suggested 5.

The overall success rate of DAIR in eradicating infection in our cohort was 89.2%. This finding is in line with our previously reported 87% success rate in treating acute PJI cases (including acute hematogenous infections) with the same protocolled approach that includes early intervention, systematic mobile parts exchange and correct “anti-biofilm” antibiotic therapy 11, 12.

The surgeon´s decision to perform additional surgeries in the early postoperative period is based on the perceived risk/benefit and often the fear of adding morbidity and the lack of obvious clinical findings frequently delays proper therapeutic intervention. Except for the *Enterococcus faecium* PJI-related death no other major medical or surgical complications related to the procedure were noted. Although it is open for debate we do not feel this complication relating specifically to the DAIR but rather an unfortunate event related to the seriousness of the infection in a frail patient with major comorbidities.

In addition to immediate complications, we were interested in finding out whether the additional surgical procedure and delay in rehabilitation would have a negative functional impact in the final outcome. After excluding patients with unsuccessful infection resolution and other unrelated confounding factors (ex. periprosthetic fracture) we found that infected patients had self-reported equivalent functional outcomes to uninfected controls. This finding that is also corroborated by other studies 13, 14, suggests that an early DAIR procedure is a “benign” procedure with no significant impact on joint function.

A major limitation of this study relates to the methodology of systematically opening the fascia as it can be argued that we may have caused bacteria to propagate from superficial layers to the joint space during the procedure. Nevertheless, we believe that our surgical technique of clearly separating deep from superficial debridement and sample gathering in the rare instances we found the fascia to be macroscopically closed is sufficiently careful and greatly minimizes this hypothetical risk. Also, the criterion used to define infection may be perceived as controversial. Still, one must consider that during the entire study period there was no universal clear definition of PJI in the acute setting. Recently, a PJI definition was proposed by the latest ICM consensus that also aims to define acute infection 15 but in our view, this definition lacks enough evidence and just recently it has been shown that the proposed thresholds may be missing a large proportion of infected cases 16.

It is our belief that a positive growth in at least one appropriately gathered deep sample together with an unfavorable clinical course is sufficiently specific to assume a PJI diagnosis. Although the required prolonged antibiotic therapy after assuming a PJI diagnosis is not without inherent complications, it is indisputable that missing and infection and letting it reach chronicity offers much bigger medical and surgical implications. Sendi at al. 17 have also shown that adopting a policy for early recognition of patients with persistent wound problems and prompt surgical treatment with DAIR leads equally high rates of success in eradicating the infection.

Although there is extensive evidence that successful total joint arthroplasty greatly increases quality of life and joint function, information on the impact of PJI are seldomly evaluated using objective measurements. Cahill et al. 18 and Helwig et al. 19 were able to confirm the negative impact of PJI on joint function and health-related quality of life. Aboltins et al. 20 prospectively collected data on over 2,000 TJA patients, of which PJI occurred in 41. Much the same as in our study, PJI cases treated with debridement and retention had similar improvements as patients without PJI in quality of life according to the SF-12 survey. The analysis however did not evaluate the potential influence of the infecting pathogen. Núñez et al. 21 evaluated 24 patients who underwent debridement and retention of the prosthesis due to an acute knee PJI and were in remission after 12 months' follow-up. Health-related quality of life was measured using WOMAC and SF-36 at baseline, 12 and 24 months after antibiotic treatment discontinuation. There was a significant improvement in all items from baseline to 48 months except for patients infected by *Staphylococcus aureus* who had significantly worse outcomes. In the present study, outcomes assessed using joint specific patient-reported outcome measures shows similar results in DAIR-treated and uneventful control patients and no significant differences were found according to the type of infecting microorganism.

## Conclusion

The results of this study seem to support our current policy of adopting a low threshold to assume a possible infection diagnosis and “aggressively” pursuing formal DAIR procedure when facing an unfavorable wound healing in the early postoperative period after total joint replacement. The results not only show it is possible to achieve high success rates in treating early infection but also suggests that this “aggressive” policy carries no significant impact on midterm functional results. As such, we rather risk overtreating a small proportion of cases in the acute stage than to increase the risk of chronic infection in a significant number of patients.

Although current criteria are still heavily punctuated by subjective clinical judgement it seems to be highly specific to select truly infected cases. In the future, objective criteria must be refined to help with decision-making but given the apparently “benign” impact of a repeated surgery, one can only speculate whether these criteria should evolve to become even more comprehensive.

## Figures and Tables

**Table 1:** Microorganisms isolated in 38 confirmed prosthetic joint infection cases

Isolated microorganisms	Overall (n=64)
***Gram positive***	45 (70.3%)
**CoN staphylococci**	21 (32.8%)
**Staphylococcus aureus**	18 (28.1%)
**Other**	
* Enterococcus spp.*	3 (4.7%)
* Streptococcus spp.*	2 (3.1%)
* Corynebacterium spp.*	1 (1.6%)
***Gram negative***	19 (29.7%)
**Enterobacteriaceae**	
* Escherichia coli*	7 (10.9%)
* Serratia spp.*	3 (4.7%)
* Klebsiella spp.*	1 (1.6%)
* Proteus spp.*	2 (3.1%)
* Citrobacter spp.*	1 (1.6%)
**Pseudomonas spp.**	4 (6.2%)
**Others**	
* Acinetobacter spp.*	1 (1.6%)
**Polymicrobial**	11 (17.2%)

CoN - coagulase negative.

**Table 2:** Demographic data of infected and control patients

	Case (n=26)	Controls (n=52)	p
**Joint**			.999
* THA*	12	24	
TKA	14	28	
**Age, years**	68.9	69.2	.880
**Gender**			.643
Female	16	32	
Male	10	20	
**ASA score**			.999
ASA 1	0	0	
ASA 2	21	42	
ASA 3	5	10	
ASA 4	0	0	
**BMI**	29.7	28.0	.070
**Follow-up, months**	42.1	43.65	.541

THA: total hip arthroplasty; TKA: total knee arthroplasty; ASA: American Society of Anesthesiologists; BMI: body mass index; DM: Diabetes Mellitus.

**Table 3:** Final functional results (HOOS/KOOS) of infected and control patients

	Case (n=26)	Controls (n=52)	P
Pain	91.2±6.0	87.3±12.6	.141
Other Symptoms	90.1±7.8	89.7±9.4	.844
Activities of day living	85.9±7.7	85.2±14.1	.826
Sports	62.65±13.3	56.5±16.4	.103
Quality of life	78.3±17.0	76.1±15.8	.569

KOOS - Knee injury and Osteoarthritis Outcome Score; HOOS - Hip Disability and Osteoarthritis Outcome Score.
